# Evaluating the risks and benefits of continuing *versus* withholding renin–angiotensin system inhibitors: a systematic review and meta-analysis with trial sequential analysis

**DOI:** 10.1016/j.bjao.2025.100405

**Published:** 2025-05-03

**Authors:** Laila Shalabi, Ahmed Ibrahim, Sofian Zreigh, Mohamed Rifai, Shrouk Ramadan, Mohamed A. Arafa, Osama M. Mustafa, Muhammad Ansab, Mohamed F. Krayem, Ibrahim Elsabbagh, Nour H. Mash’al, Salem Waleed, Matthieu Legrand

**Affiliations:** 1 Faculty of Medicine, Gharyan University, Gharyan, Libya; 2 Faculty of Medicine, Alexandria University, Alexandria, Egypt; 3 Faculty of Medicine, Ankara Yıldırım Beyazıt University, Ankara, Turkey; 4 Faculty of Medicine, Menoufia University, Shebin El Kom, Egypt; 5 Faculty of Medicine, Ain Shams University, Cairo, Egypt; 6 Faculty of Medicine, Al-Azhar University, Cairo, Egypt; 7 Faculty of Medicine, University of Jordan, Amman, Jordan; 8 Faculty of Medicine, Services Institute of Medical Sciences, Lahore, Pakistan; 9 Faculty of Medicine, University of Tripoli, Libya; 10 Department of Anesthesia and Perioperative Care, University of California, San Francisco, CA, USA

**Keywords:** ACE-inhibitors, angiotensin receptor blockers, cardiovascular complications, intraoperative hypotension, mortality, RASi, noncardiac surgery

## Abstract

**Background:**

The best perioperative management of renin–angiotensin system inhibitors (RASi) in patients undergoing noncardiac surgery has been an ongoing debate as a result of inconclusive previous studies and insufficient data for robust guidelines. Although continuation of RASi may lead to intraoperative hypotension, withholding might also cause postoperative complications. Our meta-analysis aims to explore the postoperative outcomes of strategies of RASi management before surgery by evaluating randomised clinical trials, to provide more definitive conclusions for clinical practice.

**Methods:**

We systematically searched PubMed, Scopus, Cochrane, and Web of Science until September 2024. Inclusion criteria included patients (≥18 yr) who underwent noncardiac surgery and received long-term RASi, which were either withheld or continued before surgery. Statistical analysis was conducted using R Studio version 4.4.2.

**Results:**

A total of seven RCTs with 8741 patients receiving long-term RASi before noncardiac surgery revealed no significant difference between continuation and withholding groups regarding cardiovascular complications (risk ratio [RR] 0.94, 95% confidence interval [CI] 0.80–1.09, *P*=0.41), mortality (RR 1.16, 95% CI 0.55–2.43, *P*=0.71), and acute kidney injury (RR 0.95, 95% CI 0.84–1.06, *P*=0.33). However, continuation of RASi was associated with a higher incidence of intraoperative hypotension (RR 1.33, 95% CI 1.23–1.44, *P*<0.001). Additionally, the incidence of postoperative severe hypertension (systolic BP >180 mm Hg) was significantly lower in the continuation group (RR 0.63, 95% CI 0.40–0.98, *P*<0.002).

**Conclusions:**

Continuing RASi before noncardiac surgery does not significantly impact mortality, cardiovascular complications or the risk of acute kidney injury. However, continuation is associated with an increased risk of intraoperative hypotension, and withholding with a higher risk of postoperative severe hypertension.

**Systematic review protocol:**

CRD42024605208 (PROSPERO).

Nearly 50% of people undergoing elective surgery have hypertension.^1^ These populations are at higher risk of postoperative complications.^2^ Angiotensin-converting enzyme inhibitors (ACEIs) and angiotensin receptor blockers (ARBs) are frequently prescribed for hypertension management, and particularly beneficial for patients with cardiovascular disease, diabetes, and renal disease.^3^ ACEIs lower blood pressure by preventing the synthesis of angiotensin II, a strong vasoconstrictor, and raising the vasodilator bradykinin. ARBs act by selective inhibition of angiotensin II receptor binding.^4^ Occurrence of hypotension during surgery has repeatedly been associated with postoperative complications.^5^ Several observational studies and meta-analyses, have suggested that continuation of renin–angiotensin system inhibitors (RASi) might be associated with an increased risk of intraoperative hypotension and potentially postoperative complications (e.g. acute kidney injury [AKI]).^6^, ^7^, ^8^ However, those studies are prone to residual confounders, which can introduce bias and compromise the reliability of the findings.^9^

Overall, the low quality of evidence regarding the best strategy for RASi before major surgery has led to weak and conflicting guidelines.^10^ The Canadian Cardiovascular Society's guidelines recommend avoiding therapy 24 h before surgery.^11^ The 2024 American Heart Association (AHA) and American College of Cardiology (ACC) guidelines recommend withdrawal of RASi before surgery to reduce intraoperative hypotension.^12^ In contrast, the 2024 European Society of Cardiology (ESC) guidelines do not provide a definitive recommendation.^13^

Accordingly, we conducted this meta-analysis of randomised trials to explore the impact of the strategy of RASi management on postoperative complications in patients undergoing noncardiac surgery.

## Methods

Our study adhered to the guidelines for conducting research set by the Preferred Reporting Items for Systematic Reviews and Meta-Analyses (PRISMA).^14^ The protocol was registered with the International Prospective Register of Systematic Reviews (PROSPERO; CRD42024605208).^15^

### Search strategy

On 26 September 2024, we conducted a comprehensive search across databases: PubMed, Cochrane Database, Scopus, and Web of Science, using MeSH terms: surgical, angiotensin II receptor blockers, and angiotensin-converting enzyme inhibitors. Search terms for each database are provided in detail in (Supplementary Table S1).

### Eligibility criteria

Eligible studies were English-language randomised clinical trials (RCTs) comparing continued RASi therapy *vs* its withdrawal before noncardiac surgery in adult patients >18 yr old. Outcomes assessed included postoperative cardiovascular complications, all-cause mortality, acute kidney injury (AKI), postoperative infection and sepsis, intraoperative hypotension, postoperative hypotension, postoperative hypertension, length of hospital stay, and length of intensive care unit stay. Observational studies and non-randomised articles were excluded.

### Study selection and data extraction

After removing duplicates using Mendeley, eight authors (IE, SW, MAA, SZ, NHM, OMM, MA, MFK) independently screened titles and abstracts using Rayyan. Conflicts were resolved by LS and AI, who then conducted independent full-text screenings. Seven authors (IE, SW, MAA, MR, NHM, OMM, MA, MFK) independently extracted data from each study using a standardised Google Sheet. LS and AI reviewed the extracted data to resolve any conflicts and ensure consistency. The standardised data extraction sheet included population characteristics, types of surgeries, and reported outcomes. We calculated the kappa statistic to assess inter-rater agreement between authors.^16^

### Risk of bias and certainty assessment

MR and AI independently assessed potential bias in each study using the Risk of Bias 2 (RoB 2) tool (Cochrane Collaborations), evaluating randomisation, deviations from intended interventions, absence of outcome data, measurement of the outcome, and selection of the stated results.^17^ Bias was categorised as ‘low risk’, ‘high risk’ or ‘some concerns’ with conflicts resolved through discussion. The GRADE (Grading of Recommendations Assessment, Development and Evaluation) methodology was used to assess the quality of evidence for each outcome, categorising it into one of four levels: very low, low, moderate, or high certainty.^18^

### Statistical analysis

The meta-analysis was carried out with R version 4.4.2 (R Foundation for Statistical Computing, Vienna, Austria) using a random-effects model throughout the analysis. Risk ratios (RRs) were determined for binary outcomes, whereas mean differences (MDs) were utilised for continuous ones. Each effect size was provided using a point estimate and a 95% confidence interval (CI). Two-tailed *P*-values <0.05 were considered statistically significant. Using the *I*^2^ statistic, heterogeneity was evaluated and interpreted following Cochrane's guidelines: values between 0% and 40% may suggest minimal heterogeneity, whereas values between 30% and 60% may indicate moderate heterogeneity. Considerable heterogeneity may be indicated by values between 50% and 90%.^19^

In addition, a leave-one-out sensitivity analysis was conducted to evaluate the robustness of the findings by systematically removing each study at a time and rerunning the analysis to ensure that no single study had an undue influence on the results. We could not use Egger's test to check for publication bias as we did not include 10 or more studies for any of the outcomes, which is required to get reliable results.^20^

We also performed a trial sequential analysis (TSA) to determine the necessary total sample size using the Trial Sequential Analysis software version 0.9.5.9 Beta.^21^ The analysis used an O'Brien-Fleming α-spending approach with a two-sided 5% boundary and included a futility assessment. We incorporated a 20% relative risk reduction in our analyses and applied a model-based variance heterogeneity correction for the required information size calculation. Additionally, a continuity correction factor of 0.5 was used in the TSA for intraoperative hypotension to validate the findings.

## Results

### Literature selection process and characteristics of the selection studies

**Fig 1:**
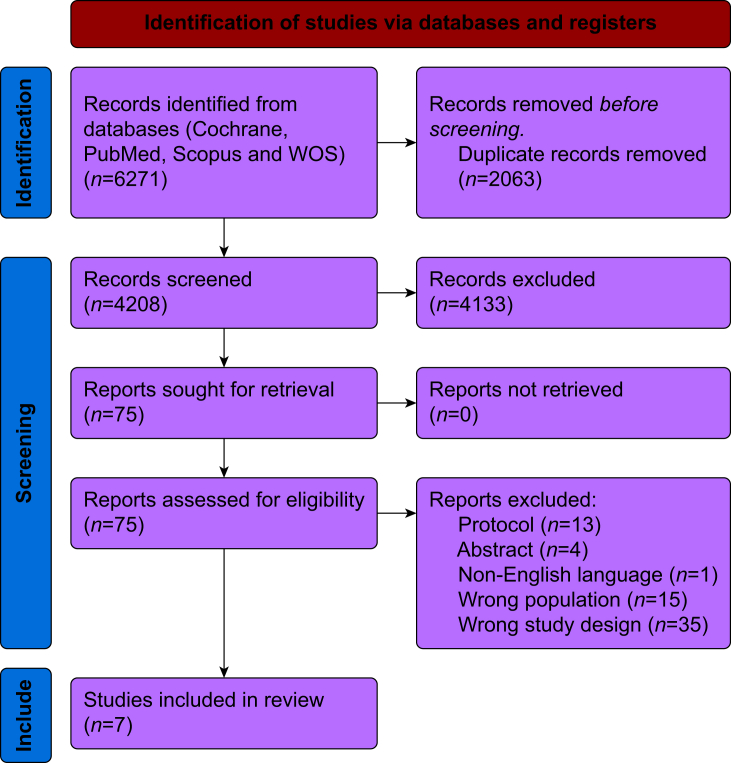
PRISMA flow chart of the screening process. WOS, Web of Science.

The initial search yielded 6271 articles. After removing duplicates, 4208 articles remained. A review of the titles and abstracts excluded 4133 articles. The remaining studies underwent full-text screening, leading to the inclusion of seven RCTs in the review.^22^, ^23^, ^24^, ^25^, ^26^, ^27^, ^28^, ^29^ The exclusion rationale for each study during the full-text screening stage is presented in (Supplementary Table S2). Inter-rater reliability between authors LS and AI, assessed using Cohen's kappa, was substantial (κ=0.75), demonstrating strong agreement in study selection. Of note, data from the POISE-3 trial were derived from two distinct publications: Marcucci and colleagues^26^ and the 2024 AKI-focused substudy.^27^ A comprehensive literature search is illustrated in the PRISMA flowchart (Fig. 1).

Seven RCTs enrolled 8741 patients who were on treatment with RASi.^22^, ^23^, ^24^, ^25^, ^26^, ^27^, ^28^, ^29^ Of these, 4365 patients had their treatment withheld before surgery and 4371 patients continued their treatment before surgery. Regarding the timing of therapy omission, one study stopped RASi at least 24 h before surgery,^22^ and four studies withheld therapy the night or day before surgery.^23^^,^^24^^,^^26^^,^^29^ One study withheld therapy at least 48 h before surgery,^28^ while Coriat and colleagues^25^ stopped captopril 12 h and enalapril 24 h before surgery. The patients' characteristics, follow-up, type of surgery, and associated comorbidities are detailed in (Table 1 and Supplementary Table S3).

**Table 1:** Baseline characteristics of the studies included in the meta-analysis. ACEIs, angiotensin-converting enzyme inhibitors; ARBs, angiotensin receptor blockers.

Study ID	Country	Study design	Intervention	Patient population		Age (yr) (range)		Male *N* (%)		Hypertension *N* (%)		Coronary artery disease *N* (%)		Follow-up
				Continuation	Withdrawal	Continuation	Withdrawal	Continuation	Withdrawal	Continuation	Withdrawal	Continuation	Withdrawal	
Ackland and colleagues 2024^22^	UK	RCT	ACEIs/ARBs	132	130	65.3–76.7	65.7–78.3	68 (51.9)	66 (51.2)	127 (96.9)	124 (97.6)	—	—	30 Days
Bertrand and colleagues 2001^23^	France	RCT	ARBs	19	18	42–94	46–90	15 (83.33)	15 (78.94)	19 (100)	18 (100)	—	—	30 Min after induction
Coriat and colleagues 1994^25^	France	RCT	Enalapril	7	11	57–73	57–77	—	—	7 (100)	11 (100)	—	—	Study ended at skin incision
			Captopril	14	19	53–83	48–84	—	—	14 (100)	19 (100)	—	—	
Legrand and colleagues 2024^28^	France	RCT	ACEIs/ARBs	1107	1115	40.6–94	40.6–94	721 (65)	730 (65)	1083 (98)	1096 (98)	183 (17)	179 (16)	28 Days
Marcucci and colleagues 2023 (POISE-3 trial)^26^	Multi countries	RCT	ACEIs/ARBs	2684	2684	51.2–88.4	51.2–88.4	2096 (56)	2075 (56)	3663 (98)	3656 (98)	1149 (31)	1116 (31)	30 Days
Substudy of POISE-3 trial^27^	Multi countries	RCT	ACEIs/ARBs	2500	2502	51.1–88.3	51.4–88.2	2050 (56)	2035 (56)	3571 (98)	3573 (98)	1121 (31)	1142 (31)	30 Days
Shiffermiller and colleagues 2018^24^	USA	RCT	ACEIs	138	137	31–96.4	31–97	68 (49)	65 (47)	112 (81)	116 (85)	19 (14)	18 (13)	48 h
Twersky 2014 and colleagues^29^	USA	RCT	ACEIs/ARBs	264	262	24.25–96.25	32.15–93.35	89 (34)	90 (34)	99 (37.5)	98 (37.4)	33/263 (13)	32/258 (12)	Postoperative

### Cardiovascular complications

**Fig 2:**
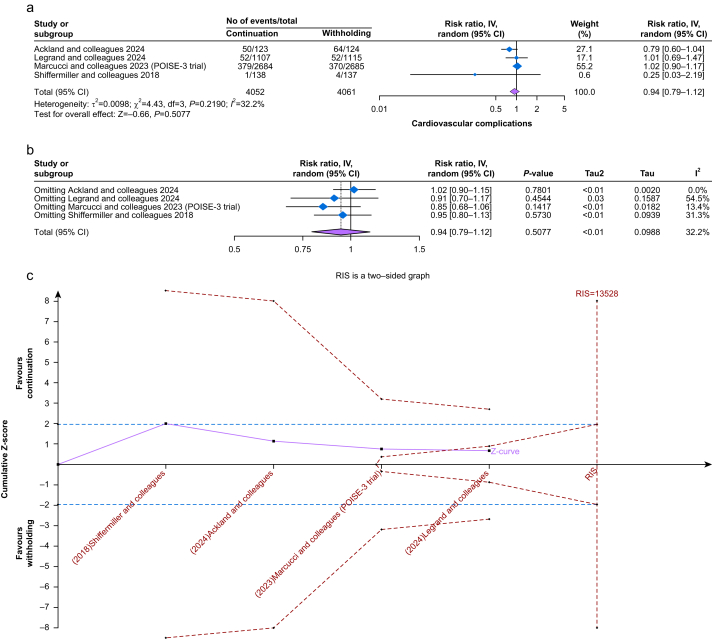
(a) Forest plot of cardiovascular complications; (b) leave-one-out sensitivity analysis of cardiovascular complications; (c) a trial sequential analysis of cardiovascular complications.

Four studies reported cardiovascular complications,^22^^,^^24^^,^^26^^,^^28^ involving 4052 patients in the continuation group and 4061 patients in the withholding group. There was no significant difference between the two groups (RR 0.94, 95% CI 0.79–1.12, *P*=0.50) with minimal heterogeneity (*I*^2^=32.2%) (Fig. 2a). The result was robust on sensitivity analysis (Fig. 2b), and the sample size was sufficient to cross the futility boundary on TSA (Fig. 2c). The certainty of evidence was high (Supplementary Table S4). Detailed definitions of cardiovascular complications for each study are provided in Supplementary Table S5.

### Acute kidney injury

**Fig 3:**
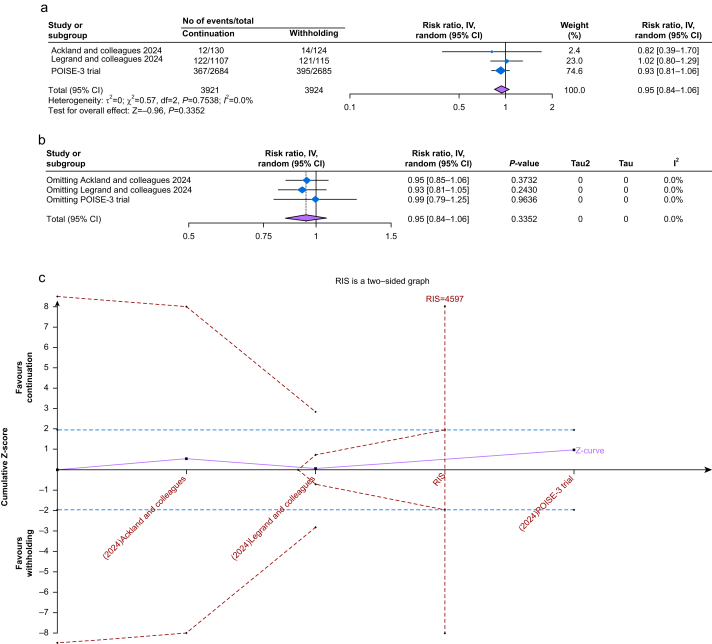
a) Forest plot of acute kidney injury; (b) leave-one-out sensitivity analysis of acute kidney injury; (c) a trial sequential analysis of acute kidney injury.

Three studies reported AKI,^22^^,^^27^^,^^28^ with 3737 patients in the continuation group and 3741 patients in the withholding group. There was no significant difference between the two groups (RR 0.95, 95% CI, 0.84–1.06, *P*=0.33) with no heterogeneity (*I*^2^=0%) (Fig. 3a). The result was robust on sensitivity analysis (Fig. 3b). The sample size was sufficient to cross the futility boundary on TSA (Fig. 3c). The certainty of evidence was high (Supplementary Table S4).

### All-cause mortality

**Fig 4:**
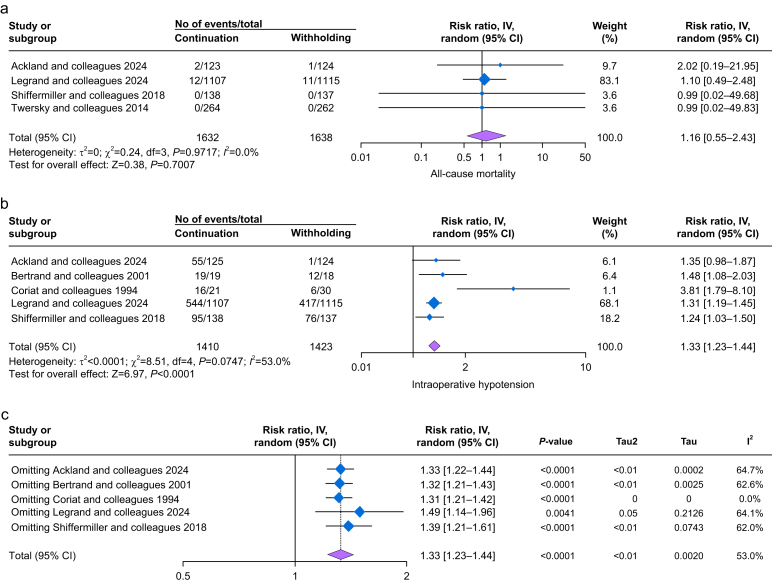
(a) Forest plot of all-cause mortality; (b) forest plot of intraoperative hypotension; (c) leave-one-out sensitivity analysis of intraoperative hypotension.

Four studies reported mortality,^22^^,^^24^^,^^28^^,^^29^ with 1632 patients in the continuation group and 1638 patients in the withholding group. There was no significant difference between the two groups (RR 1.16, 95% CI 0.55–2.43, *P*=0.70) with no heterogeneity (*I*^2^=0%) (Fig. 4a). Of these studies, two RCTs involving 801 patients reported no mortality.^24^^,^^29^ The result was robust on sensitivity analysis (Supplementary Fig. S1). TSA showed insufficient evidence to detect a difference (Supplementary Fig. S2). The certainty of evidence was moderate (Supplementary Table S4).

### Postoperative infection and sepsis

The outcome was evaluated in two studies^22^^,^^28^ with 1230 patients in the continuation group and 1239 in the withholding group. The two groups had no significant difference between the two groups (RR 0.92, 95% CI 0.62–1.36, *P*=0.68) with no heterogeneity (*I*^2^=0%) (Supplementary Fig. S3).

### Intraoperative hypotension

Five studies reported intraoperative hypotension,^22^, ^23^, ^24^, ^25^^,^^28^ with 1410 patients in the continuation group and 1423 patients in the withholding group. The continuation group was significantly associated with a higher risk of intraoperative hypotension (RR 1.33, 95% CI 1.23–1.44, *P*<0.001) with a moderate heterogeneity (*I*^2^=53%) (Fig. 4b). The result was robust on sensitivity analysis. Additionally, heterogeneity was significantly reduced (*I*^2^=0%) after removing (Coriat and colleagues^25^) (Fig. 4c). TSA demonstrated conclusive evidence for hypotension risk reduction with RASi withdrawal (Supplementary Fig. S4). The certainty of evidence was low (Supplementary Table S4).

### Postoperative hypotension

Postoperative hypotension was evaluated in two studies.^22^^,^^24^ There was no significant difference between the two groups (RR 1.40, 95% CI 0.61–3.21, *P*=0.42) with considerable heterogeneity (*I*^2^=66.6%) (Supplementary Fig. S5).

### Postoperative hypertension

**Fig 5:**
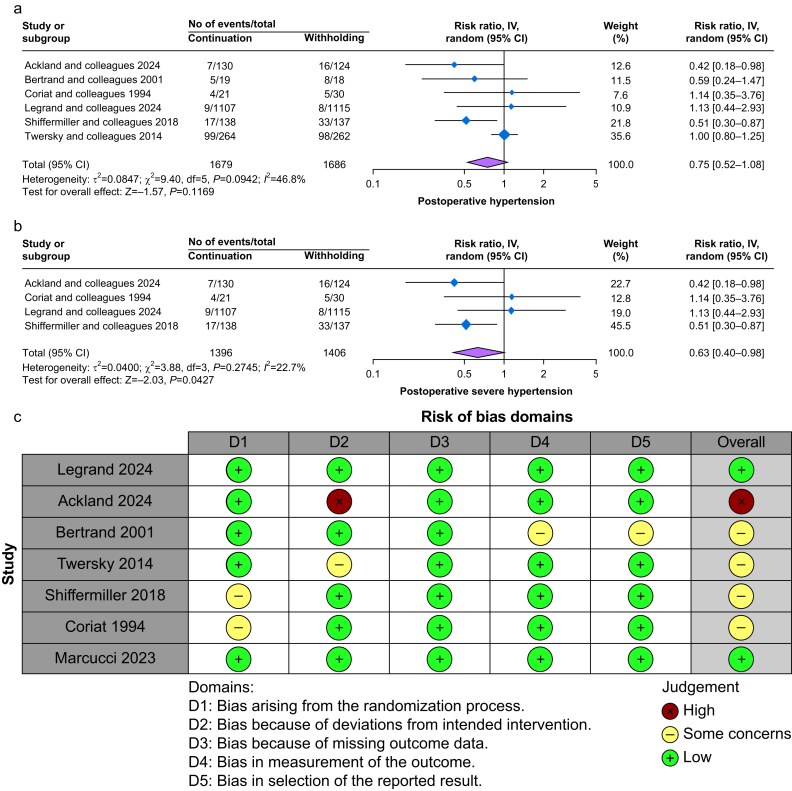
(a) Forest plot of postoperative hypertension; (b) forest plot of postoperative severe hypertension; (c) quality assessment of risk of bias for the included trials.

Postoperative hypertension was evaluated in six studies,^22^, ^23^, ^24^, ^25^^,^^28^^,^^29^ with 1679 patients in the continuation group and 1686 patients in the withholding group. There was no significant difference between the two groups (RR 0.75, 95% CI 0.52–1.08, *P*=0.11) with a moderate level of heterogeneity (*I*^2^=46.8%) (Fig. 5a). A leave-one-out sensitivity analysis showed removing Shiffermiller and colleagues^24^ yielded a significant difference, suggesting this study influenced the initial non-significant results (Supplementary Fig. S6). TSA showed insufficient evidence to detect a difference (Supplementary Fig. S7). The certainty of evidence was low (Supplementary Table S4).

Additionally, subgroup analysis was performed based on studies that reported hypertension crises (MAP >130 mm Hg or systolic BP >180 mm Hg).^22^^,^^24^^,^^25^^,^^28^ The continuation group was significantly associated with a lower risk of postoperative severe hypertension (systolic BP >180 mm Hg) (RR 0.63, 95% CI 0.40–0.98, *P*<0.002) (Fig. 5b). However, sensitivity analysis revealed lack of robustness, as the exclusion of Ackland and colleagues^22^ or Shiffermiller and colleagues^24^ rendered the results insignificant (Supplementary Fig. S8). TSA showed insufficient evidence to detect a difference (Supplementary Fig. S9). The certainty of evidence was moderate (Supplementary Table S4).

### Length of hospital and intensive care unit stays (days)

Legrand and colleagues^28^ and Ackland and colleagues^22^ assessed hospital and intensive care unit (ICU) stays. There was no significant difference between the two groups for the ICU stay (MD −0.24, 95% CI −1.68 to 1.120, *P*=0.74) (Supplementary Fig. S10) or hospital stay (MD −0.24, 95% CI −0.33 to 0.33, *P*=1.00) (Supplementary Fig. S11).

### Risk of bias

Based on the assessment using the RoB 2 tool, among seven RCTs reviewed, only two studies (Legrand and colleagues^28^ and Marcucci and colleagues^26^) demonstrated a ‘low’ risk of bias in all domains. Twersky and colleagues^29^ had a ‘low’ risk of bias in most domains, but ‘some concerns’ in Domain 2. Ackland and colleagues^22^ had a ‘low’ bias except for Domain 2. Bertrand and colleagues^23^ had ‘some concerns’ in Domains 4 and 5. Shiffermiller and colleagues^24^ and Coriat and colleagues^25^ had some concerns in Domain 2 and a high risk in Domain 4 (Fig. 5c). The Cohen's kappa (between author MR and AI) was 0.71, indicating substantial agreement.

## Discussion

This systematic review and meta-analysis aimed to evaluate the efficacy and safety of continuing *vs* withholding RASi therapy in the perioperative setting of noncardiac surgery. The analysis included seven RCTs, involving a total of 8741 patients. The continuation of RASi presents a complex balance of risks and benefits. Whereas continuation is linked to an increased risk of intraoperative hypotension, it does not significantly affect postoperative complications such as mortality, cardiovascular complication, or AKI. However, continuation may offer protection against postoperative severe hypertension (hypertensive crisis). The 2024 ACC and AHA guidelines suggest withholding RASi 24 h before high-risk noncardiac surgery to reduce intraoperative hypotension, which is consistent with our findings showing that continuation of RASi therapy is linked to increased intraoperative hypotension.^12^ It appears, however, that these episodes of hypotension are being appropriately treated and do not translate into a higher risk of complications. This observation aligns with the existing literature, which has not established a definitive association between intraoperative hypotension and subsequent major morbidity or mortality outcomes.^9^^,^^30^^,^^31^ Furthermore, numerous case reports and reviews have documented significant intraoperative hypotension, especially within the first 30 min after induction,^32^ in patients who continue RASi therapy during general or neuraxial anaesthesia.^25^^,^^33^ Conversely, some observational studies indicate that withholding these medications for >10 h before surgery can result in better blood pressure control, irrespective of the specific drug used.^32^ In the meta-analysis by Hollmann and colleagues,^8^ which specifically focused on noncardiac data, a 30% relative risk increase in intraoperative hypotension was identified when RASi therapy was continued on the morning of surgery. This indicates that patients who maintained their RASi treatment were more likely to experience intraoperative hypotension compared with those who withheld their medications. These findings further support our results.

Although withholding RASi showed no overall difference in postoperative hypertension, some studies suggest a potential increased risk of severe hypertension.^22^^,^^24^ Notably, consistent with the POISE-3 trial, our analysis found that this increased risk did not significantly impact overall postoperative complications, mortality, or cardiovascular events.^26^^,^^27^

Our analysis provides evidence that continued RASi therapy does not increase the risk of AKI, which may contradict previous reports.^7^^,^^34^ Our analysis indicated no significant difference in mortality between the two study groups, a finding consistent with Turan and colleagues.^35^ However, Railton and colleagues^36^ reported a significant association between perioperative RASi inhibitor therapy and 30-day postoperative mortality in patients undergoing vascular surgery.

Additionally, our analysis supplied by TSA provides evidence that the continuation of RASi therapy does not elevate the risk of cardiovascular complications, a finding that aligns with the results of previous studies.^26^

### Strengths and limitations

Previous meta-analyses were, however, largely driven by results from observational studies.^8^^,^^34^ Results from observational studies are exposed to unmeasured confounders and can result in inaccurate estimation between an exposure and an outcome. The goal of randomisation is to minimise the impact of confounders by ensuring that both known and unknown confounders are evenly distributed between the treatment groups. In this meta-analysis, we only included randomised trials, and we reduced the risk of selection bias, therefore increasing the reliability of the findings. Our meta-analysis represents a comprehensive and up-to-date evaluation of this topic, incorporating all relevant RCTs to achieve high-quality evidence. Furthermore, the results of the TSA offer conclusions on primary outcomes, such as AKI and cardiovascular complications. Nevertheless, some limitations must be acknowledged. The inclusion of open-label studies introduces a risk of bias; however, we attempted to address this by conducting sensitivity analysis where applicable. Additionally, while we observed some heterogeneity in certain outcomes, we conducted sensitivity analyses to ensure the robustness of our results. A notable limitation across several studies on this topic is the use of different definitions for hypotensive thresholds, variable drug dosages, the timing of RASi withdrawal, and the types of anaesthesia used. Furthermore, the recruitment period ranged from the early 1990s to the present day, which may have influenced the observed outcomes because of changes in clinical practices and guidelines over time. Finally, the underrepresentation of high-risk populations, such as patients with heart failure or chronic kidney disease, indeed limits the generalisability of this meta-analysis to these subgroups. Of note, large databases show that these subpopulations represent <5% of patients undergoing major noncardiac surgery.^37^^,^^38^

### Conclusions

In conclusion, in this meta-analysis of randomised trials, continuing RASi during the preoperative period does not significantly affect cardiovascular complications, mortality, or risk of AKI. Continuing RASi was associated with a higher risk of intraoperative hypotension but a lower risk of postoperative severe hypertension.

## Authors’ contributions

Conceptualisation and study question: LS, AI, SZ, ML

Design and methodology: LS, AI, ML

Development of search strategy: LS, AI, MAA

Acquisition of data: all authors

Development of statistical analytical plan: AI, SZ, ML

Statistical analyses: AI, SZ

Drafting the manuscript: LS, AI, SZ, SR, MAA, OMM, ML

Critical revision of the manuscript: all authors

Visualisation: AI, SZ, MR

Supervision: AI, ML

Project administration: LS

Contributed significantly to the reported work, participated in drafting, revising, or critically reviewing the manuscript, provided final approval for the version to be published, agreed on the journal to which the manuscript has been submitted, and accepted responsibility for all aspects of the work: all authors

## Funding

National Institutes of Health (R01-GM151494-01, R01DK139484-01) to ML.

## Declaration of interest

ML received consulting fees from Alexion, Radiometer, Viatris, and La Jolla.
