# Supplementary material for: Evaluating the risks and benefits of continuing versus withholding renin–angiotensin system inhibitors: a systematic review and meta-analysis with trial sequential analysis
Source: BJA Open. 2025 May 3;14:100405. doi: 10.1016/j.bjao.2025.100405 (PMC12104715; doi:10.1016/j.bjao.2025.100405)
Supplement: Multimedia component 1 [file mmc1.docx]

Supplementary File

1. **Figure S1:** Leave-one-out sensitivity analysis of all-cause mortality.
2. **Figure S2:** A trial sequential analysis of All-cause mortality.
3. **Figure S3:** Forest plot of Post-operative Infection and Sepsis.
4. **Figure S4:** A trial sequential analysis of Intraoperative hypotension.
5. **Figure S5:** Forest plot of Post-operative hypotension.
6. **Figure S6:** Leave-one-out sensitivity analysis of Post-operative hypertension.
7. **Figure S7:** A trial sequential analysis of Post-operative hypertension.
8. **Figure S8:** Leave-one-out sensitivity analysis of Post-operative severe hypertension.
9. **Figure S9:** A trial sequential analysis of Post-operative severe hypertension.
10. **Figure S10:** Forest plot of intensive care unit (ICU) stay (day).
11. **Figure S11:** Forest plot of Length of hospital stay (day).
12. **Table S1:** Search term used for each database.
13. **Table S2:** Exclusion rationale for studies during full-text screening stage.
14. **Table S3:** Types of Surgery of the Studies Included in the Meta-Analysis.
15. **Table S4:** Summary of evidence certainty using GRADE.
16. **Table S5**: Definitions of cardiovascular complications outcome in Included Studies.


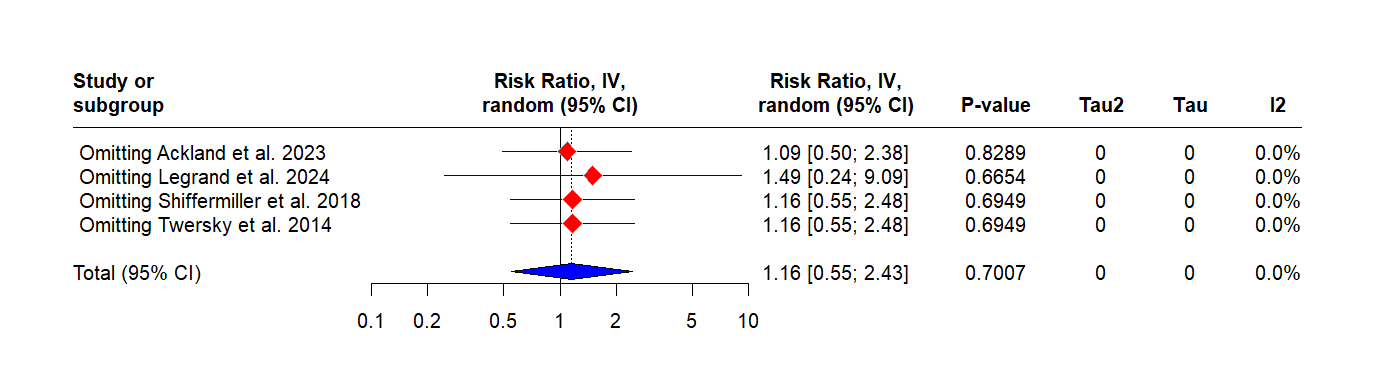


**Figure S1:** Leave-one-out sensitivity analysis of all-cause mortality.


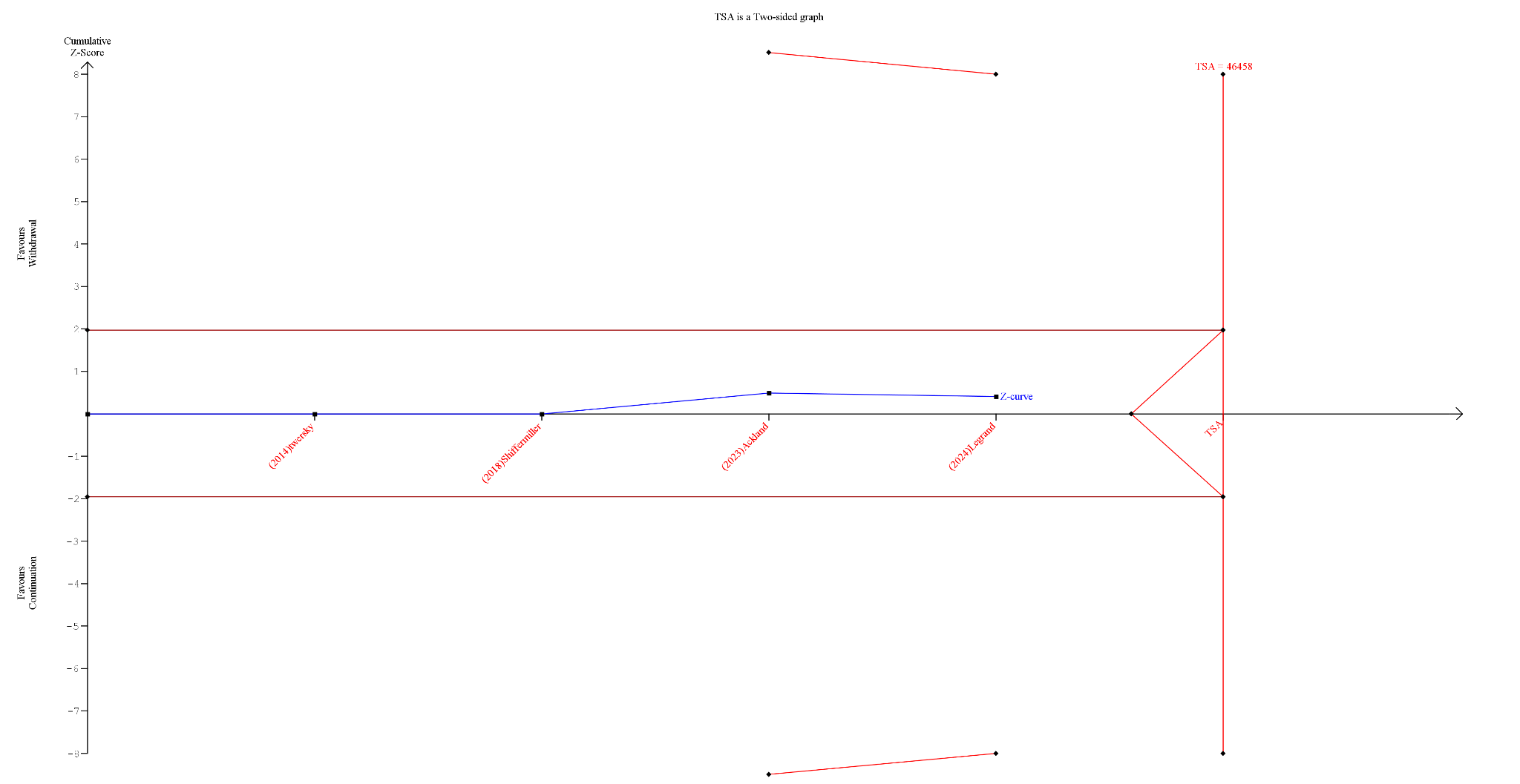


**Figure S2:** A trial sequential analysis of all-cause mortality.

TSA of withdrawal or continuation of RASi for all-cause mortality using 80% power, 5% significance, to detect the specified effect size. The required sample size was 46458 (vertical red line). The cumulative Z-curve (blue line with small black squares representing each trial) did not cross the traditional boundary (horizontal red line), the superiority boundary (trial sequential monitoring boundary) (concave red line), or the futility boundary (convex red line), indicating a lack of evidence (false negative).

**
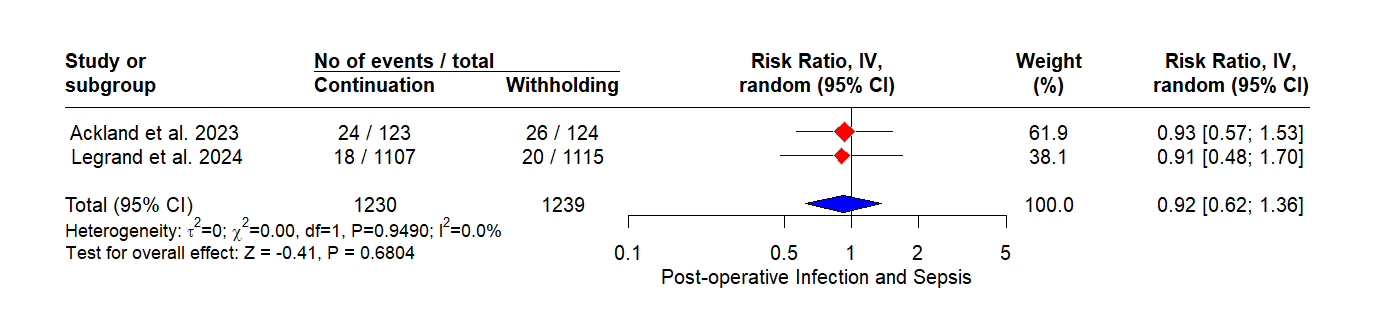
**

**Figure S3:** Forest plot of Post-operative Infection and Sepsis.


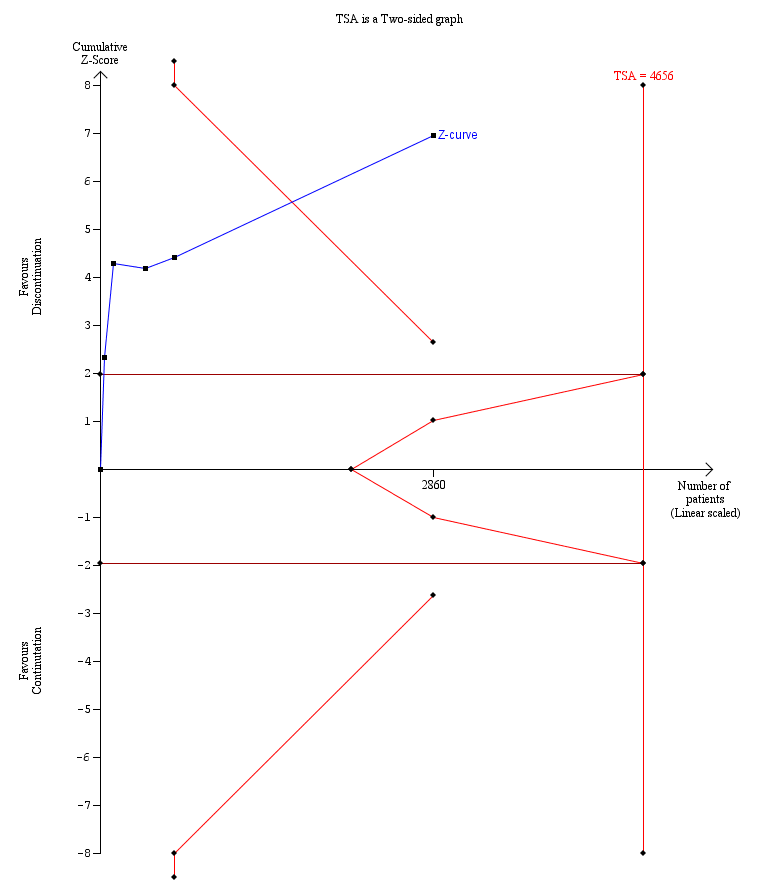


**Figure S4:** A trial sequential analysis of Intraoperative hypotension.

TSA of withdrawal or continuation of RASi for cardiovascular events using 80% power, 5% significance, to detect the specified effect size. The required sample size was 4656 (vertical red line). Sixty-one percent of the required sample size was accumulated. The cumulative Z-curve (blue line with small black squares representing each trial) crossed both the traditional boundary (horizontal red line) and the superiority boundary (trial sequential monitoring boundary) (concave red line), indicating evidence is sufficient (true positive).

**
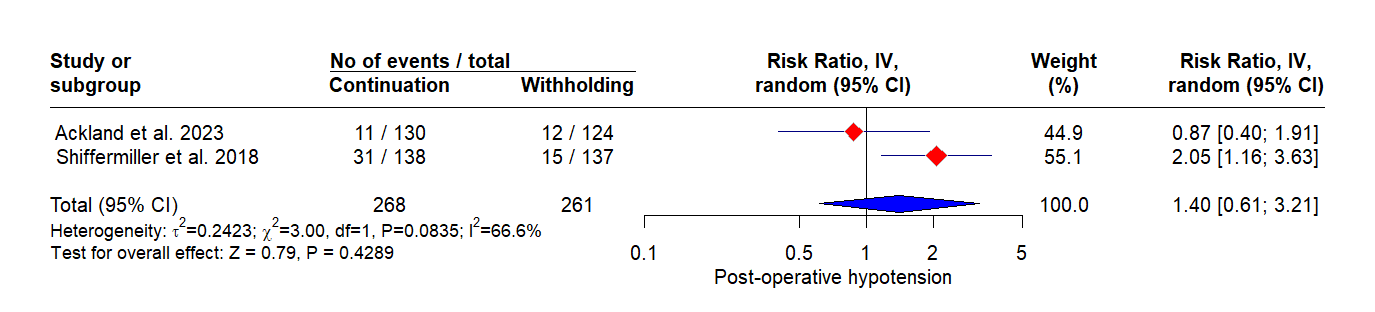
**

**Figure S5:** Forest plot of Post-operative hypotension.

**
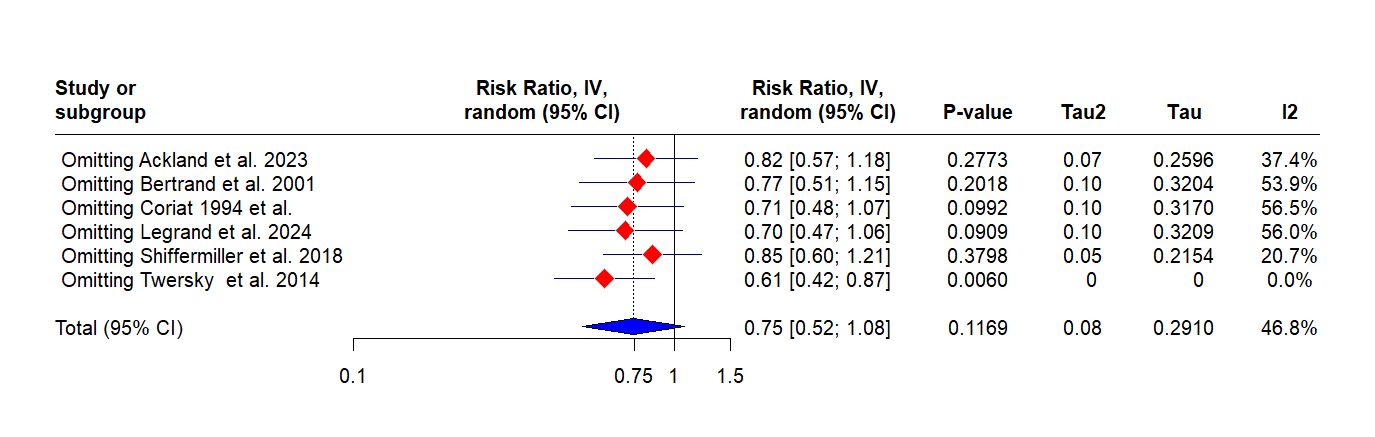
**

**Figure S6:** Forest plot of Post-operative hypertension.


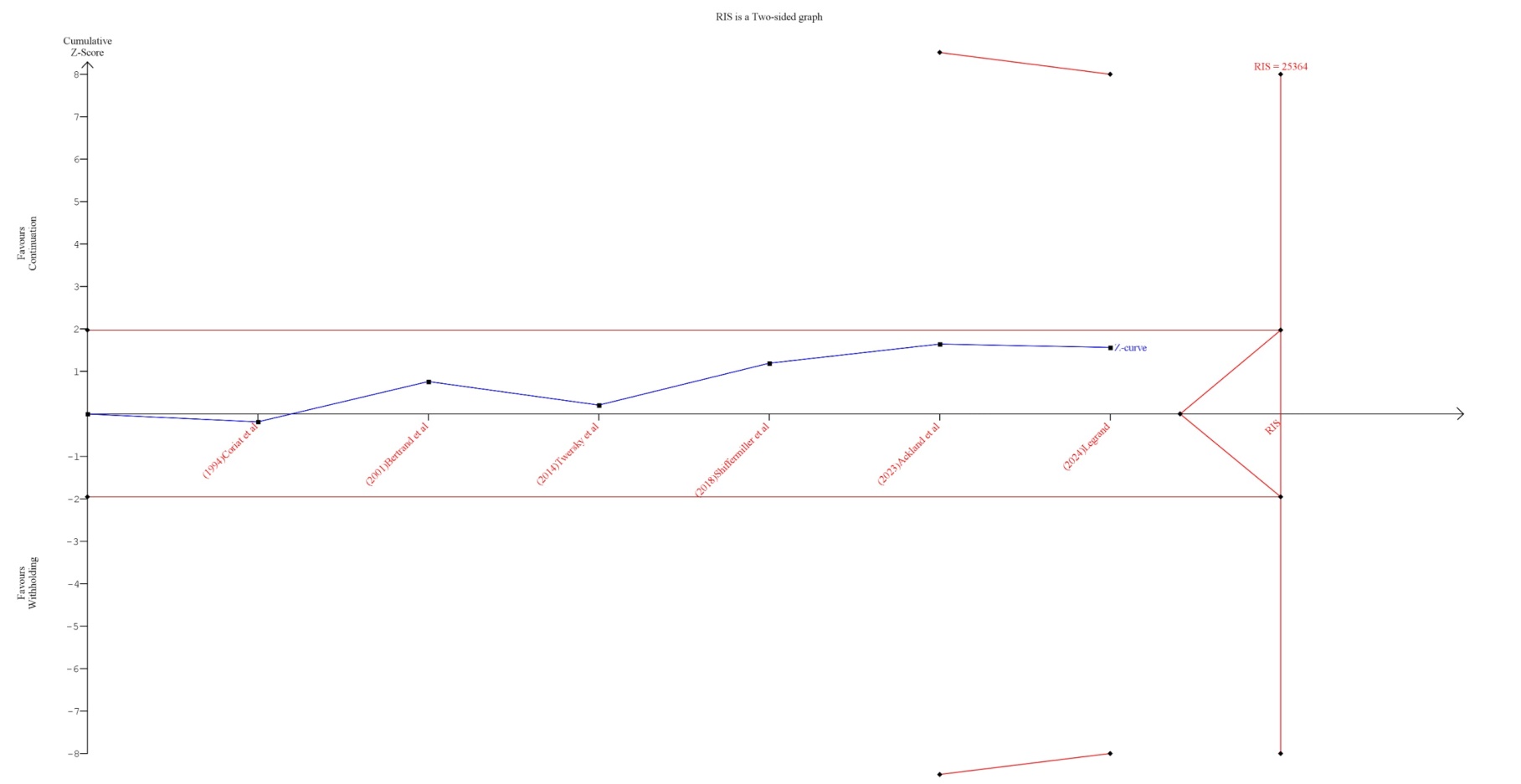


**Figure S7:** A trial sequential analysis of Post-operative hypertension.

TSA of withdrawal or continuation of RASi for post-operative hypertension using 80% power, 5% significance, to detect the specified effect size. The required sample size was 25364 (vertical red line). The cumulative Z-curve (blue line with small black squares representing each trial) did not cross the traditional boundary (horizontal red line), the superiority boundary (trial sequential monitoring boundary) (concave red line), or the futility boundary (convex red line), indicating a lack of evidence (false negative).

**
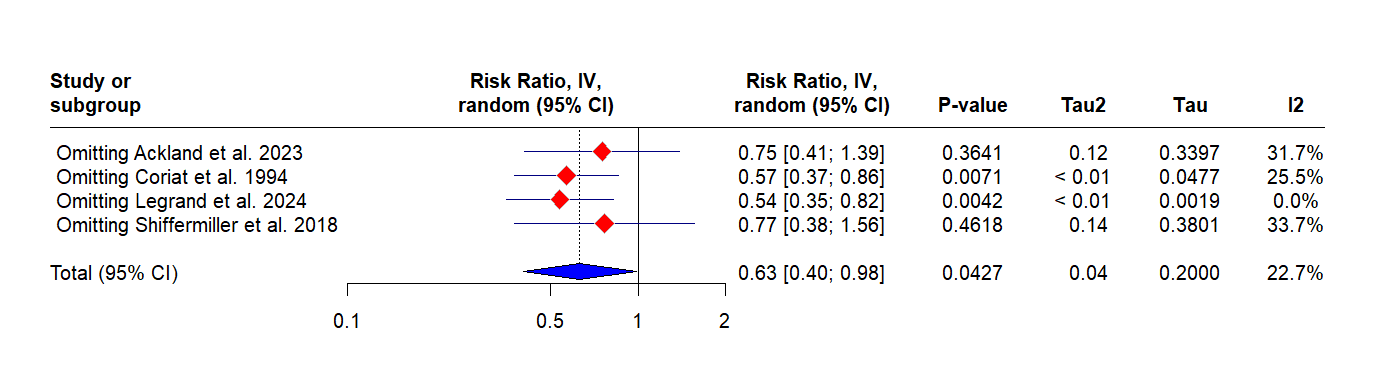
**

**Figure S8:** Forest plot of Post-operative severe hypertension.


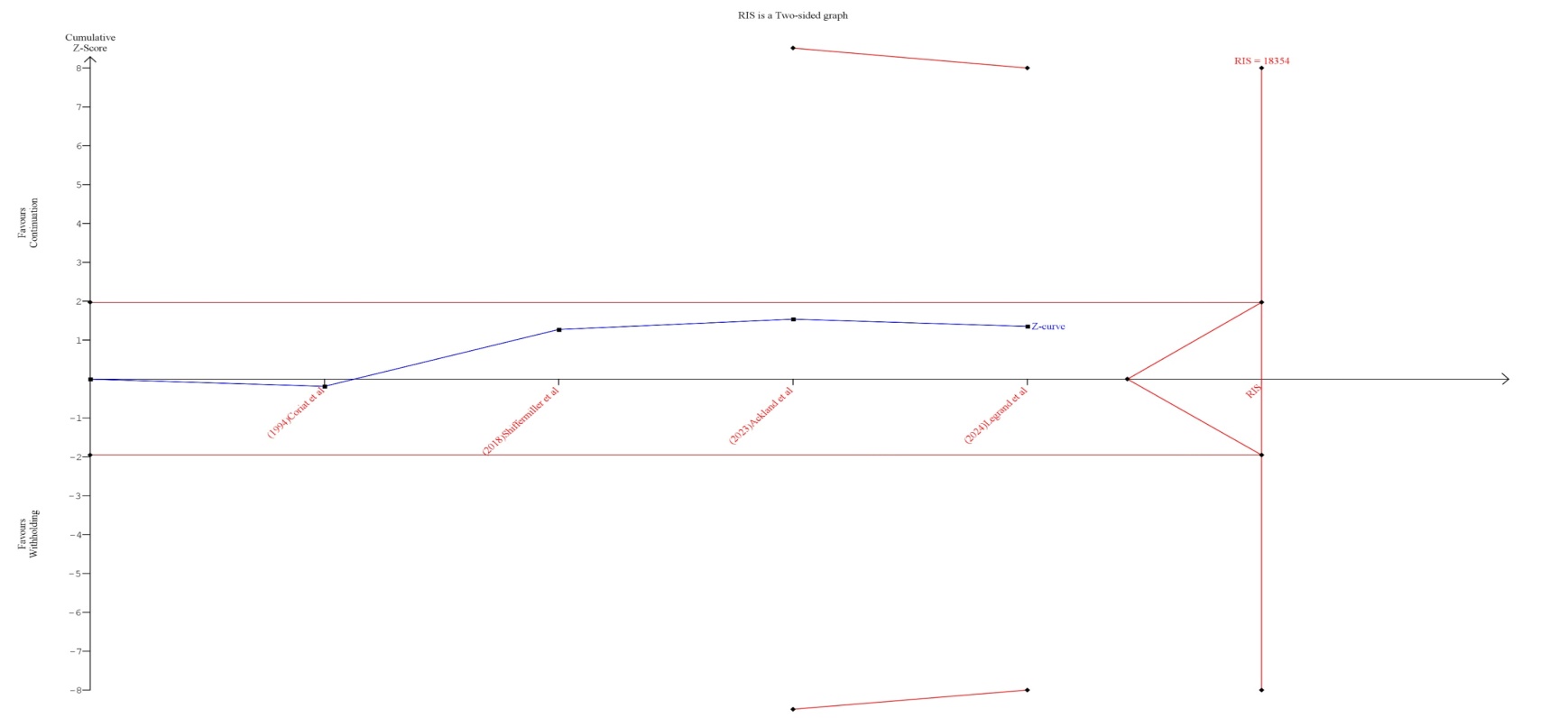


**Figure S9:** A trial sequential analysis of Post-operative severe hypertension.

TSA of withdrawal or continuation of RASi for severe post-operative hypertension using 80% power, 5% significance, to detect the specified effect size. The required sample size was 18354 (vertical red line). The cumulative Z-curve (blue line with small black squares representing each trial) did not cross the traditional boundary (horizontal red line), the superiority boundary (trial sequential monitoring boundary) (concave red line), or the futility boundary (convex red line), indicating a lack of evidence (false negative).


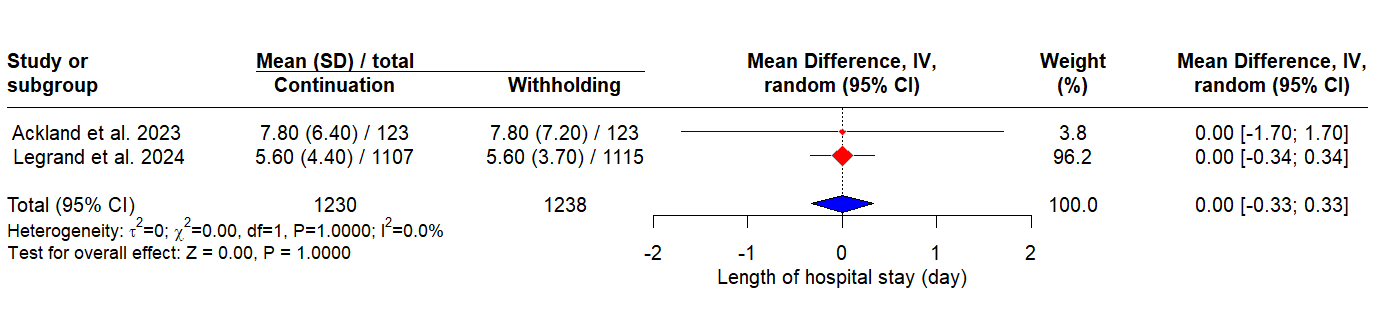


**Figure S10:** Forest plot of intensive care unit (ICU) stay (day).

**Figure S11:** Forest plot of Length of hospital stay (day). **
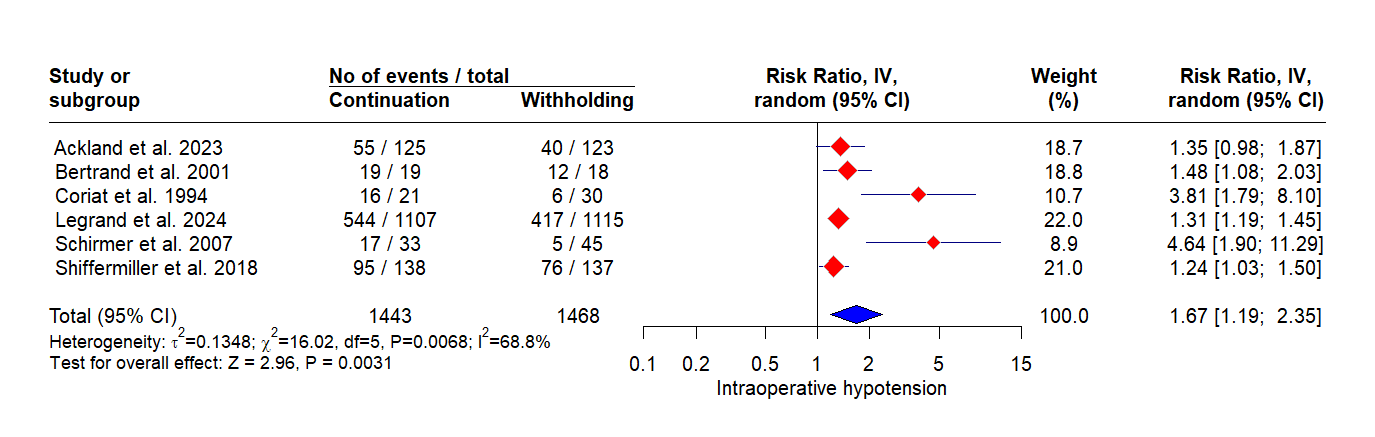
**

| Database | Search Term | Number of articles |
| --- | --- | --- |
| Pubmed | (operative[Title/Abstract] OR surgical[Title/Abstract] OR surgery[Title/Abstract]) AND ('Angiotensin II Receptor Blocker'[Title/Abstract] OR 'Angiotensin Receptor Blocker'[Title/Abstract] OR 'Angiotensin II Receptor Antagonist'[Title/Abstract] OR 'Angiotensin Receptor Antagonist'[Title/Abstract] OR 'Angiotensin-Converting Enzyme'[Title/Abstract] OR 'angiotensin-converting enzyme inhibitor'[Title/Abstract] OR 'Angiotensin Converting Enzyme Inhibitor'[Title/Abstract] OR 'Angiotensin-Converting Enzyme Antagonist'[Title/Abstract] OR 'ACE Inhibitor'[Title/Abstract] OR 'ACE-I'[Title/Abstract] OR ARB[Title/Abstract] OR ACEI[Title/Abstract] OR 'Renin-Angiotensin System Inhibitor'[Title/Abstract] OR RASI[Title/Abstract]) | 2,081 |
| Scopus | TITLE-ABS-KEY ( ( operative OR surgical OR surgery ) AND ( 'angiotensin AND ii AND receptor AND blocker' OR 'angiotensin AND receptor AND blocker' OR 'angiotensin AND ii AND receptor AND antagonist' OR 'angiotensin AND receptor AND antagonist' OR 'angiotensin-converting AND enzyme' OR 'angiotensin-converting AND enzyme AND inhibitor' OR 'angiotensin AND converting AND enzyme AND inhibitor' OR 'angiotensin-converting AND enzyme AND antagonist' OR 'ace AND inhibitor' OR 'ace-i' OR arb OR acei OR 'renin-angiotensin AND system AND inhibitor' OR rasi ) ) | 193 |
| WOS | TS=((operative OR surgical OR surgery) AND ('Angiotensin II Receptor Blocker' OR 'Angiotensin Receptor Blocker' OR 'Angiotensin II Receptor Antagonist' OR 'Angiotensin Receptor Antagonist' OR 'Angiotensin-Converting Enzyme' OR 'angiotensin-converting enzyme inhibitor' OR 'Angiotensin Converting Enzyme Inhibitor' OR 'Angiotensin-Converting Enzyme Antagonist' OR 'ACE Inhibitor' OR 'ACE-I' OR ARB OR ACEI OR 'Renin-Angiotensin System Inhibitor' OR RASI)) | 3,046 |
| Cochrane | ((operative OR surgical OR surgery) AND ('Angiotensin II Receptor Blocker' OR 'Angiotensin Receptor Blocker' OR 'Angiotensin II Receptor Antagonist' OR 'Angiotensin Receptor Antagonist' OR 'Angiotensin-Converting Enzyme' OR 'angiotensin-converting enzyme inhibitor' OR 'Angiotensin Converting Enzyme Inhibitor' OR 'Angiotensin-Converting Enzyme Antagonist' OR 'ACE Inhibitor' OR 'ACE-I' OR ARB OR ACEI OR 'Renin-Angiotensin System Inhibitor' OR RASI) in Title Abstract Keyword - (Word variations have been searched) | 951 |

**Table S1:** Search term used for each database.

| **Number** | **Title** | **Exclusion cause** |
| --- | --- | --- |
| 1 | [Angiotensin converting enzyme inhibitors in controlled hypotension during spinal surgery]. | Wrong population |
| 2 | A comparison of continuation of one-quarter dose and full-dose ACEIs /ARBs effect on incidence of intraoperative hypotension in patients undergoing non-cardiac surgery, a randomized controlled study | Wrong population |
| 3 | A comparison of continuation of one-quarter dose and full-dose ACEIs/ARBs on hemodynamic effects in patient undergoing non-cardiac surgery: a RCT study | Wrong population |
| 4 | A randomized clinical trial comparing preoperative continuation versus cessation of angiotensin converting enzyme inhibitors and angiotensin receptor blockers in patients undergoing elective, noncardiac surgery at a major tertiary hospital | Protocol |
| 5 | ACE inhibition does not exaggerate the blood pressure decrease in the early phase of spinal anaesthesia | Single arm |
| 6 | Angiotensin axis blockade, acute kidney injury, and perioperative morbidity in patients undergoing colorectal surgery A retrospective cohort study | Observational study |
| 7 | Angiotensin II Receptor Blockers but Not Angiotensin-Converting Enzyme Inhibitors Are Associated With a Reduced Risk of Acute Kidney Injury After Major Surgery | Observational study |
| 8 | Angiotensin Receptor Blockers and Angiotensin-Converting Enzyme Inhibitors Have No Significant Relationship With Postoperative Arthrofibrosis After Shoulder Arthroscopy. | Observational study |
| 9 | Association between peri-operative angiotensin-converting enzyme inhibitors and angiotensin-2 receptor blockers and acute kidney injury in major elective non-cardiac surgery: a multicentre, prospective cohort study | Observational study |
| 10 | Association between preoperative renin-angiotensin system inhibitor use and postoperative acute kidney injury risk in patients with hypertension | Abstract |
| 11 | Association between renin-angiotensin-aldosterone system blockers and postoperative atrial fibrillation in patients with mild and moderate left ventricular dysfunction | Wrong population |
| 12 | Association of renin angiotensin antagonists with adverse perioperative events in patients undergoing elective orthopaedic surgery: a case-control study | Wrong study design |
| 13 | Beneficial effect of angiotensin converter enzyme inhibitors on left ventricular deformation in patients with hematologic malignancies after bone marrow transplantation | Abstract |
| 14 | Chronic angiotensin-converting enzyme inhibitor or angiotensin receptor blocker therapy combined with diuretic therapy is associated with increased episodes of hypotension in noncardiac surgery | Wrong population |
| 15 | Chronic arterial hypertension and nocturnal non-dipping predict postinduction and intraoperative hypotension: A secondary analysis of a prospective study. | Secondary analysis |
| 16 | Chronic Use of Angiotensin Converting Enzyme Inhibitors and/or Angiotensin Receptor Blockers is Not Associated With Stroke After Noncardiac Surgery: A Retrospective Cohort Analysis | Observational study |
| 17 | Clinical consequences of withholding versus administering renin-angiotensin-aldosterone system antagonists in the preoperative period | Review |
| 18 | Comparison of effects of telmisartan versus valsartan on post-induction hypotension during noncardiac surgery: a prospective observational study | Observational study |
| 19 | Continuation of Renin-Angiotensin-Aldosterone Inhibitors Does Not Impact Renal Function Among Patients Undergoing Percutaneous Nephrolithotomy | Review |
| 20 | Continuation or stopping antihypertensives in patients undergoing non-cardiac surgery | Protocol |
| 21 | Continuing versus withholding angiotensin receptor blocker (ARB)/calcium channel blocker (CCB) combination tablets during perioperative periods in patients undergoing minor surgery: a single-blinded randomized controlled trial | Wrong population |
| 22 | Discontinuing drugs before surgery | Review |
| 23 | Effect of a Perioperative Hypotension-Avoidance Strategy Versus a Hypertension-Avoidance Strategy on the Risk of Acute Kidney Injury: a Clinical Research Protocol for a Substudy of the POISE-3 Randomized Clinical Trial | Protocol |
| 24 | Effect of continuing angiotensin-converting enzyme inhibitors or angiotensin II receptor blockers on the day of surgery on myocardial injury after non-cardiac surgery: A retrospective cohort study | Observational study |
| 25 | Effect of different use strategies of angiotensin-converting enzyme inhibitors or angiotensin II receptor blockers on perioperative hypotension and adverse cardiovascular events in older patients undergoing noncardiac surgery: a prospective, multicenter, randomized, double-blind, controlled trial | Protocol |
| 26 | Effect of fluvastatin on renal end points in the Assessment of Lescol in Renal Transplant (ALERT) trial | Wrong population |
| 27 | Effect of perioperative continuous or interrupted administration of ACEI or ARB on postoperative delirium in elderly patients | Protocol |
| 28 | Effect of pre-operative discontinuation of angiotensin-converting enzyme inhibitors or angiotensin II receptor antagonists on intra-operative arterial pressures after induction of general anesthesia. | Wrong population (insufficient information on surgery type) |
| 29 | Effect of pre-transplantation use of angiotensin-converting enzyme inhibitor or angiotensin receptor blocker in kidney transplant recipients-propensity score-matched analysis. | Observational study |
| 30 | Effects of antihypertensive drugs on surgical outcomes of breast reconstruction: a nationwide population-based claim study | Observational study |
| 31 | Effects of preoperative continuation or withdrawal of ARBs on postoperative complications in elderly patients undergoing noncardiac surgery 30 days after surgery: a prospective randomized controlled trial | Protocol |
| 32 | Effects of withholding ACEIs/ARBs or not in the morning on intraoperative blood pressure in hypertensive patients undergoing non-cardiac surgery: a randomized controlled trial | Protocol |
| 33 | Frequency of Intraoperative Hypotension After the Induction of Anesthesia in Hypertensive Patients with Preoperative Angiotensin-converting Enzyme Inhibitors | Single arm |
| 34 | Hemodynamic effects of angiotensin system inhibitors in patients undergoing elective joint arthroplasty | Abstract |
| 35 | Hemodynamic effects of withholding vs. continuing angiotensin II receptor blockers on the day of prone positioning spinal surgery in elderly patients | Wrong study design |
| 36 | Impact of Angiotensin Receptor Blocker Use on Overall Survival Among Patients Undergoing Resection for Pancreatic Cancer | Wrong population |
| 37 | Impact of angiotensin system inhibitors on esophageal cancer survival | Wrong population |
| 38 | Impact of Renin-Angiotensin System Inhibitors Continuation on Outcome After Major Surgery | Protocol |
| 39 | Influence of angiotensin-converting enzyme inhibitors on hypotension after anesthetic induction: is the preoperative discontinuation of this drug necessary? | Observational study |
| 40 | Influence of converting enzyme inhibition on isoflurane-induced hypotension for cerebral aneurysm surgery. | Wrong population |
| 41 | Influence of Renin-Angiotensin System Inhibitors on Postoperative Delirium in Patients With Pulmonary Arterial Hypertension: A Secondary Analysis of a Retrospective Cohort Study | Secondary analysis |
| 42 | Influence of whether ARB was discontinued before operation on hemodynamics in elderly patients undergoing spinal surgery in prone position | Protocol |
| 43 | Intra- and postoperative relative angiotensin II deficiency in patients undergoing elective major abdominal surgery | Single arm |
| 44 | Long-term mortality benefit of renin-angiotensin system inhibitors in patients with chronic limb-threatening ischemia undergoing vascular intervention | Wrong population |
| 45 | Management of angiotensin inhibitors during the perioperative period | Protocol |
| 46 | Perioperative management of hypertension | Abstract |
| 47 | Perioperative of ACEI/ARB induce acute kidney injury in major abdominal surgery: a single-center randomized controlled study | Single arm |
| 48 | Perioperative Quality Initiative consensus statement on postoperative blood pressure, risk and outcomes for elective surgery | Wrong study design |
| 49 | Predictors of hypotension during anesthesia induction in patients with hypertension on medication: a retrospective observational study | Observational study |
| 50 | Preoperative activation of the Reninâ€“Angiotensin system and myocardial injury in noncardiac surgery: post Hoc Analysis of the SPACE randomised controlled Trial | Secondary analysis |
| 51 | Preoperative administration of angiotensin-converting enzyme inhibitors | Not English |
| 52 | Preoperative hemodynamic Changes after Continuing or discontinuing Regular Angiotensin Converting Enzyme Inhibitors before Cataract Surgery a Comparative Study | Protocol |
| 53 | Preoperative N-terminal pro-B-type natriuretic peptide and myocardial injury after stopping or continuing renin-angiotensin system inhibitors in noncardiac surgery: a prespecified analysis of a phase 2 randomised controlled multicentre trial | Secondary analysis |
| 54 | Preoperative Renin-Angiotensin System Antagonists Intake and Blood Pressure Responses during Ambulatory Surgical Procedures: A Prospective Cohort Study | Observational study |
| 55 | Prognostic Impact of Renin-Angiotensin Inhibitors in Patients with Bladder Cancer Undergoing Radical Cystectomy | Wrong study design |
| 56 | Prognostic Impact of Renin-Angiotensin System Blockade on Renal Cell Carcinoma After Surgery | Wrong population |
| 57 | Protecting the Heart with Cardiac Medication in Patients with Left Ventricular Dysfunction Undergoing Major Noncardiac Vascular Surgery | Wrong population |
| 58 | Randomized controlled trial of Stopping Perioperative Angiotensin-II Converting Enzyme inhibitors and/or Angiotensin Receptor Blockers in major noncardiac surgery | Protocol |
| 59 | Renin-angiotensin system blocker use and the risk of acute kidney injury after colorectal cancer surgery: a population-based cohort study | Observational study |
| 60 | Renoprotective effect of early inhibition of the renin-angiotensin system in renal transplant recipients | Observational study |
| 61 | Should ARB/CAB combination tablets be continued or withheld preoperatively? | Wrong study design |
| 62 | Stopping perioperative angiotensin II converting enzyme inhibitors and/or angiotensin receptor blockers in major noncardiac surgery | Protocol |
| 63 | The association of withholding or continuing angiotensin-converting enzyme inhibitors or angiotensin 2 receptor blockers on acute kidney injury after non-cardiac surgery | Observational study |
| 64 | The Canadian ACE-inhibitor trial to improve renal outcomes and patient survival in kidney transplantation--study design | Wrong study design |
| 65 | The Effect of Renin-Angiotensin System Inhibitors in Patients Undergoing Pancreatic Cancer Resection | Observational study |
| 66 | The effects of perioperative angiotensin converting enzyme inhibitors and angiotensin receptor blockers on acute kidney injury in major elective non-cardiac surgery. A multicentre, prospective cohort study | Observational study |
| 67 | The use of renin angiotensin aldosterone system inhibitors may be associated with decreased mortality after cancer surgery | Wrong population |
| 68 | Withholding versus Continuing Angiotensin-converting Enzyme Inhibitors or Angiotensin II Receptor Blockers before Noncardiac Surgery: an Analysis of the Vascular events In noncardiac Surgery patIents cOhort evaluatioN Prospective Cohort | Observational study |

**Table S2**: Exclusion rationale for studies during full-text screening stage.

| **Study ID** | **Type of surgery** |
| --- | --- |
| Ackland et al. 2023^22^ | Joint replacement, vascular, gastrointestinal surgery |
| Bertrand et al. 2001^23^ | Vascular Surgery |
| Coriat et al. 1994^25^ | Vascular surgery |
| Legrand et al. 2024^28^ | Abdominal, thoracic, vascular, urological, orthopaedic, pelvic, neurosurgical, liver |
| POISE-3 trial^26, 27^ | General, Orthopaedic, Vascular, Urological, Gynaecological, Thoracic, Spinal, Plastic, Low risk surgery |
| Shiffermiller et al. 2018^24^ | Non-cardiac & non-vascular surgeries |
| Twersky et al. 2014^29^ | Ambulatory and same day surgery |

**Table S3:** Types of Surgery of the Studies Included in the Meta-Analysis

| **Certainty assessment** | | | | | | | **Summary of findings** | | | | |
| --- | --- | --- | --- | --- | --- | --- | --- | --- | --- | --- | --- |
| **Participants (studies) Follow-up** | **Risk of bias** | **Inconsistency** | **Indirectness** | **Imprecision** | **Publication bias** | **Overall certainty of evidence** | **Study event rates (%)** | | **Relative effect (95% CI)** | **Anticipated absolute effects** | |
|  |  |  |  |  |  |  | **With [withhold]** | **With [continuation]** |  | **Risk with [withhold]** | **Risk difference with [continuation]** |
| **Cardiovascular complications** | | | | | | | | | | | |
| 8113 (4 RCTs) | not serious | not serious | not serious | not serious | none | ⨁⨁⨁⨁ High | 490/4061 (12.1%) | 482/4052 (11.9%) | **RR 0.94** (0.79 to 1.12) | 490/4061 (12.1%) | **7 fewer per 1,000** (from 25 fewer to 14 more) |
| **All-cause mortality** | | | | | | | | | | | |
| 3270 (4 RCTs) | not serious | not serious | not serious | serious^c^ | none | ⨁⨁⨁◯ Moderate | 12/1638 (0.73%) | 14/1632 (0.86%) | **RR 1.16** (0.55 to 2.43) | 12/1638 (0.73%) | **1 more per 1,000 (from 3 fewer to 10 more)** |
| **Acute kidney injury** | | | | | | | | | | | |
| 7478 (3 RCTs) | not serious | not serious | not serious | not serious | none | ⨁⨁⨁⨁ High | 530/3741 (14.2%) | 501/3737 (13.4%) | **RR 0.95** (0.84 to 1.06) | 530/3741 (14.2%) | **7 fewer per 1,000** (from 23 fewer to 9 more) |
| **Intraoperative Hypotension** | | | | | | | | | | | |
| 2833 (5 RCTs) | serious^a^ | serious^b^ | not serious | not serious | none | ⨁⨁◯◯  Low | 551/1423 (38.7%) | 729/1410 (51.7%) | **RR 1.33** (1.23 to 1.44) | 551/1423 (38.7%) | **128 more per 1,000 (from 89 more to 170 more)** |
| **Post-operative hypertension** | | | | | | | | | | | |
| 3365 (6 RCTs) | serious^a^ | serious^b^ | not serious | serious^c^ | none | ⨁⨁◯◯  Low | 168/1686 (10%) | 141/1679 (8.4%) | **RR 0.75** (0.52 to 1.08) | 168/1686 (10%) | **25 fewer per 1,000 (from 48 fewer to 8 more)** |
| **Post-operative severe hypertension** | | | | | | | | | | | |
| 2802 (4 RCTs) | serious^a^ | not serious | not serious | serious^c^ | none | ⨁⨁⨁◯  Moderate | 62/1406 (4.4%) | 37/1396 (2.7%) | **RR 0.63** (0.40 to 0.98) | 62/1406 (4.4%) | **17 more per 1,000 (from 4 more to 30 more)** |

**Table S4:** Summary of evidence certainty using GRADE.

CI: confidence interval; MD: mean difference; RR: risk ratio.

**Explanations:**

a. most of the included RCTs showed a high or an unclear overall risk of bias.

b. A moderate or high level of heterogeneity.

c. A wide confidence interval that does not exclude the appreciable harm/benefit.

| **Study** | **Definition of Cardiovascular Complications** |
| --- | --- |
| Ackland et al., 2023^22^ | This study included a composite of reported cardiovascular outcomes, including myocardial injury, myocardial infarction, acute heart failure, and stroke. |
| Legrand et al., 2024^28^ | Cardiovascular complications were defined as acute myocardial infarction, arterial or venous thrombosis, stroke, acute pulmonary oedema, cardiogenic shock, acute severe hypertensive crisis, or de novo cardiac arrhythmia requiring therapeutic intervention. |
| Marcucci et al., 2023^26^ | The composite outcome included vascular death, nonfatal myocardial injury after non-cardiac surgery (MINS), stroke, and cardiac arrest. |
| Shiffermiller et al., 2018^24^ | A composite of acute coronary syndrome, acute heart failure, or new-onset arrhythmia. |

**Table S5:** Definitions of Cardiovascular Complications Outcome in Included Studies.
